# Reversed Septal Curvature Is Associated with Elevated Troponin Level in Hypertrophic Cardiomyopathy

**DOI:** 10.1155/2020/8821961

**Published:** 2020-11-28

**Authors:** Renata Rajtar-Salwa, Tomasz Tokarek, Paweł Petkow Dimitrow

**Affiliations:** ^1^Department of Cardiology and Cardiovascular Interventions, University Hospital, Jakubowskiego 2 St., 30-688 Krakow, Poland; ^2^2nd Department of Cardiology, Institute of Cardiology, Jagiellonian University Medical College, 31-501 Krakow, Poland

## Abstract

The aim of study was to compare patients with hypertrophic cardiomyopathy divided according to septal configuration assessed in a 4-chamber apical window. The study group consisted of 56 consecutive patients. Reversed septal curvature (RSC) and non-RSC were diagnosed in 17 (30.4%) and 39 (69.6%) patients, respectively. Both RSC and non-RSC groups were compared in terms of the level of high-sensitivity troponin I (hs-TnI), NT-proBNP (absolute value), NT-proBNP/ULN (value normalized for sex and age), and echocardiographic parameters, including left ventricular outflow tract gradient (LVOTG). A higher level of hs-TnI was observed in RSC patients as compared to the non-RSC group (102 (29.2-214.7) vs. 8.7 (5.3-18) (ng/l), *p* = 0.001). A trend toward increased NT-proBNP value was reported in RSC patients (1279 (367.3-1186) vs. 551.7 (273-969) (pg/ml), *p* = 0.056). However, no difference in the NT-proBNP/ULN level between both groups was observed. Provocable LVOTG was higher in RSC as compared to non-RSC patients (51 (9.5-105) vs. 13.6 (7.5-31) (mmHg), *p* = 0.04). Furthermore, more patients with RSC had prognostically unfavourable increased septal thickness to left LV diameter at the end diastole ratio. Patients with RSC were associated with an increased level of hs-TnI, and the only trend observed in this group was for the higher NT-proBNP levels. RSC seems to be an alerting factor for the risk of ischemic events. Not resting but only provocable LVOTG was higher in RSC as compared to non-RSC patients.

## 1. Introduction

Monitoring of biomarkers including troponin (Tn) and N-terminal pro-B-type NT-pronatriuretic peptide (NT-proBNP) might be utilized in the clinical evaluation, management, and prognosis of patients with hypertrophic cardiomyopathy (HCM) [1]. Recently reported papers have studied the importance of very short time synchronization in the sampling of echocardiographic parameters and cardiac biomarkers in HCM [2, 3]. The strategy of these studies was based on performing transthoracic echocardiography (TTE) and evaluation of hs-troponin I and NT-proBNP as close to each other as possible. Such tactic guarantees that currently detected ischemia is related to actual myocardial functional/dynamic status with provoked left ventricular outflow tract gradient (LVOTG) as an equivalent. This protocol is crucial to obtain reliable results of this unstable parameter. Provocation of higher LVOTG is strongly associated with increased myocardial oxygen consumption inducing ischemia [4, 5]. The strategy of simultaneous measurement of the relationship between gradient provocation and ischemia induction was confirmed in invasive studies [4, 5]. Thus, the potential relationship between current ischemia (probably frequently repeated in past history) and echocardiographic stabile septal configuration predisposing to myocardial ischemia (possibly even during all the life span) should be evaluated. This echocardiographic abnormal configuration is reversed septal curvature (RSC) visualized in 4-chamber apical view. Importantly, a pathomorphological study revealed that RSC occurs more frequently in young than in elderly victims of sudden cardiac death (SCD) [6]. This fact might suggest that majority of patients with RSC configuration died prematurely probably due to ischemia-provoked ventricular fibrillation. In a previous study, RSC was considered as a risk factor for SCD in HCM [7]. Thus, we sought to compare both RSC and non-RSC groups in terms of the biomarker level and echocardiographic parameters, including LVOTG.

## 2. Materials and Methods

The group of adult 56 consecutive patients with HCM was enrolled to study analysis after several exclusions. The study protocol was approved by the local ethics committee board (Bioethics Committee of Jagiellonian University KBET/119/B/2017). All included patients provided written informed consent to participate in the study. The study protocol conforms to the ethical guidelines of the 1975 Declaration of Helsinki with later amendments. All analysed patients fulfilled diagnostic criteria for HCM [8]. The standard definition in adult patients was used to recognize HCM with TTE parameter evaluation ([8]. Patients with (*n* = 40) or without (*n* = 16) prescribed treatment (newly diagnosed patients referred to our ambulatory clinic) were examined by TTE with LVOT gradient provocation by two natural stimuli (orthostatic test and the Valsalva test) [8–10]. Exclusion criteria were as follows: ST-segment or non-ST-segment elevation myocardial infarction (current or previous), significant coronary stenosis in recent coronary angiography, previous alcohol septal ablation, dilated LV cavity and decreased LV contractibility, atrial fibrillation, and arterial hypertension. Only patients with LV ejection fraction > 50% were enrolled. Furthermore, renal failure is a typical extracardiac factor related to TnI elevation. To overcome potential bias related to this factor, patients with elevated serum creatinine levels resulting in estimated glomerular filtration rate < 60 ml/min/1.73 m^2^ were excluded from the study group.

We performed genetic analysis with positive findings only in part of our patients [11]. According to Bos et al. [12], univariate and multivariate analyses demonstrated echocardiographic RSC, age at diagnosis < 45 years, maximal LVWT ≥ 20 mm, family history of HCM, and family history of SCD to be positive predictors of the positive genetic test while hypertension was a negative predictor. All our patients had at least one above-mentioned predictor of the positive genetic test, and the negative predictor was excluded.

Several storage diseases were excluded using the following criteria. Fabry disease was excluded by the absence of clinical signs and symptoms: acroparesthesis, angiokeratomas, anhydrosis, and renal dysfunction—proteinuria and lower filtration rate. Finally, in suspected patients, enzymatic analysis was performed. Pompe or Danon diseases were excluded by the following exclusive criteria: (1) patients below 18 years of age, (2) muscle weakness, skeletal myopathy, and (3) elevation of creatine kinase. Apart from storage disease, patients treated with steroids or tacrolimus were excluded.

**Figure 1:**
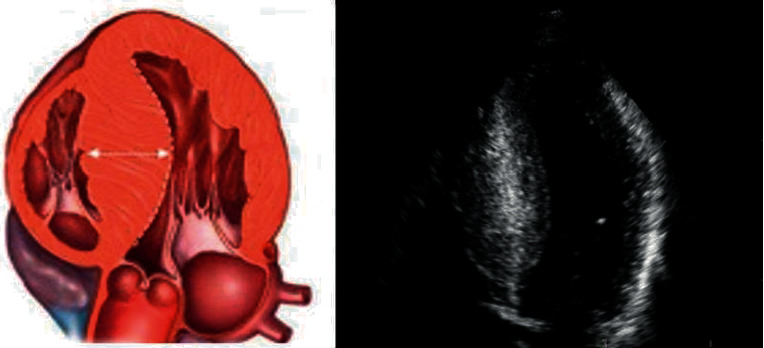
Reversed septal curvature. Predominant midseptal convexity toward left ventricle cavity; crescent-shaped cavity.

Only patients with coronary microvessel disease, which might be common in HCM at any age, were enrolled in the study group (inclusion criteria: normal/near-normal coronary arteries or no indication of coronary arteriography). Almost all patients with CCS III class (*n* = 9/10) and one with CCS IV (*n* = 1/1) underwent coronary angiography. Most of the patients have not undergone coronary angiography (young, without angina, and without risk factors for CAD—especially without diabetes mellitus). The probability of CAD was low; thus, there was no clinical indication to perform coronary angiography. Diabetes mellitus is associated with silent ischemia from epicardial coronary arteries; thus, it was necessary to exclude painless macrovascular disease [13]. All included patients were compared according to septal curvature assessed in 4-chamber apical view. There were 39 patients in the subgroup with nonreversed septal curvature (non-RSC) and 17 patients with RSC (Figure 1).

In the non-RSC subgroup, there were basic septal, neutral (no convex/no concave toward LV cavity), and no apical variant. Additionally, we analysed the ratio of septal thickness divided by LV diameter at end diastole moment (STD/LVEDD). This ratio was calculated using echocardiographic parameters measured in long-axis parasternal view. STD was measured also in short-axis parasternal view (if STD was greater than 2 mm (few cases), the patient was excluded) [7]. Patients were also excluded if maximal LV thickness was located out of the septal sector (2 patients). These patients after comprehensive analysis were rediagnosed as Fabry disease. This parameter is linked to an unfavourable prognosis when the result is above 0.5 [7]. Furthermore, both RSC and non-RSC groups were compared in terms of the level of high-sensitivity troponin I (hs-TnI), NT-proBNP (absolute value), and NT-proBNP/ULN (value normalized for sex and age). A cut-off value of 19 ng/l was used according to the manufacturer's instructions (biometry VIDAS® high-sensitivity troponin I). This value represents the 99th percentile of a presumably healthy population. High-sensitivity troponin tests were performed with the use of the VIDAS high-sensitivity troponin I (TNHS). The test is capable of measuring cardiac troponin I concentration in the range of 4.9-40,000.00 pg/ml (ng/1) without the need for dilution. The level of NT-proBNP was evaluated with the use of an Elecsys proBNP II Cobas e601 system. The test is able to measure the NT-proBNP concentration in the range of 5–35,000 pg/ml without the need for dilution. The NT-proBNP levels were presented as absolute values and standardized to sex and age on the basis of the manufacturer's guidelines (http://www.rochecanada.com/content/dam/roche_canada/en_CA/documents/package_inserts/ProBNPII-04842464190-EN-V9-CAN.pdf). The value of NT-proBNP above the 95th percentile for age and gender (the ULN) was considered abnormal. Therefore, the results were expressed as the ratio of the NT-proBNP to age- and sex-matched ULN. Rate > 1.0 was considered to be abnormal [14]. This standardization of NT-proBNP provides a normal distribution of data, whereas absolute values were distributed abnormally. Thus, there was no need for logarithmic transformation for artificial calculation. Standard descriptive statistics were used. Quantitative variables were expressed as the mean and standard deviation (SD) (normally distributed data—Kolmogorov-Smirnov) or median and interquartile range (IQR) (abnormal distribution of data). Categorical variables were presented with counts and as percentages (Fisher exact or Chi-squared test). Differences between two groups were evaluated using an independent *t*-test or Mann-Whitney-Wilcoxon test dependent on distribution of data. The results were considered significant at *p* value of 0.05 or lower. All statistical analyses were performed using STATISTICA v 13 software (StatSoft, Inc., Kraków, Poland).

## 3. Results

The study group included 30 men and 26 women. The mean age of all patients was 45 ± 6 (years). Most of them was on treatment—31 (55%) on beta-blockers, 19 (34%) on verapamil, and 6 (11%) on diuretics. Baseline clinical data is presented in Table 1. Almost all patients with CCS III class (*n* = 9/10) and one with CCS IV (*n* = 1/1) underwent coronary angiography. RSC was related to CCS III/IV. A higher level of hs-TnI was observed in RSC patients as compared to the non-RSC group. A trend toward increased NT-proBNP value was reported in RSC patients. However, no difference in the NT-proBNP/ULN level was observed between both groups (Table 2). In TTE parameters, the RSC group was characterized by a higher value of provocable LVOTG as compared to non-RSC patients; however, similar resting LVOT was observed. In M-mode measurements, STD was greater and LVEDD was smaller in RSC vs. non-RSC; thus, STD/LVEDD > 0.5 was more frequently reported in the RSC subgroup (Table 2). There was a higher rate of nsVT, syncope, and family history of SCD in the RSC group (Table 2).

Patients with non-RSC had more than twice lower risk of SCD according to the ESC calculator (3.09 (1.9-4.76) vs. 8.83 (4.37-12.35) (%/5 years), *p* = 0.002) (Table 2). In addition, in the RSC subgroup, majority of patients were at high risk of SCD according to the ESC risk calculator (>6%/5 years vs. <4%/5 years: 53% vs. 18%, *p* = 0.007).

## 4. Discussion

The results of our analysis suggest that patients with RSC were associated with an increased level of hs-TnI. Additionally, a trend was observed in this group for the higher NT-proBNP but not in NT-proBNP levels. RSC seems to be an alerting, morphological sign related to ischemic events. Only provocable LVOTG was higher in RSC as compared to non-RSC patients. Furthermore, more patients with RSC had a prognostically unfavourable increased STD/LVEDD ratio [7]. In a pioneered paper by Lever et al., the RSC with crescent LV cavity size was detected only in younger HCM patients [15]. Analysing the relationship between age and RSC in a pathomorphological study [6], advanced abnormality in myocardial architecture (strongly related to reversed RSC) was common in patients who died at a mean age of 25 years. In contrast, most of the patients who were older than 65 years when they died had a normal circular unit surrounding the LV cavity as a morphological marker of non-RSC. Hypothetically, SCD may be related to RSC via repetitive ischemic episodes. To evaluate the cumulative effect of both increased septal hypertrophy and decreased LV cavity size, we used the proportion of two TTE parameters measured at end diastole (the ratio of septal thickness to left ventricular diastolic diameter (STD/LVEDD)). In searching for other morphological substrates that culminate in the STD/LVEED ratio as SCD risk factors, we hypothesised that a small LV cavity might be a predictor of SCD because it is often linked with several risk factors (from different guidelines) for SCD, syncope [16], abnormal blood pressure response to exercise [17], and massive LV hypertrophy [18]. A narrow LV cavity easily predisposes to hypotensive mediated syncope during LV low input-low output failure triggered by tachycardia [19]. It was postulated that the combination of low filling volume and nonsustained ventricular tachycardia (nsVT) might lead to sudden depression of cardiac output with severe hypotension [19]. Thus, ventricular fibrillation might be triggered as the cumulative effect of myocardial ischemia (due to hypotension mediated reduction of coronary perfusion) and electrical instability. In a recently published study [20], authors have defined two subgroups of HCM patients: the first subgroup had RSC morphology with more fibrosis, but less resting obstruction, whereas the second subgroup presented non-RSC configuration with more frequent obstruction occurrence and less fibrosis. The authors postulated the need for further investigation comparing these subgroups to search for risk factors for SCD. This reasonable conception had been partially verified in the year 2005 in a small-scale study [7] where RSC had been recognized as a risk cofactor for SCD. In our opinion, the lack of provoked obstruction in all included patients might limit observed results. The European Society of Cardiology (ESC) risk score of SCD recommended the use of maximal-provocable LVOT gradient rather than the resting one [8]. Importantly, in this study, we have documented that the maximized value of provocable LVOT gradient was associated with an increased level of high-sensitivity troponin [4]. In another study [20], the positive troponin test corresponded with a higher ESC risk score of SCD as compared to patients with a negative troponin test estimated according to the SCD calculator (including provocable LVOT gradient as a component) (6.38% ± 4.17% vs. 3.81% ± 3.23%, *p* < 0.05). Furthermore, the authors stated that TTE examination should be performed as close as possible to the moment of enrollment. It created unfavourable delay for biomarker assessment. In contrast, we stressed that TTE and biomarker sampling/measurement must be close to each other as possible [2, 3]. The strategy of simultaneous measurement of the relationship between gradient provocation and ischemia induction was also effectively used in the invasive study [4, 5]. The pathophysiological mechanism behind ischemia in RSC is still elusive. However, RSC patients have a higher volume of postcontrast hyperenhancement in MRI study suggesting a greater amount of ischemical necrosis [19]. The RSC generating catenoid shape of the septum might lead to septal immobility because a ventricular segment with a net zero curvature would develop internal tension with no isometric contraction. Adjacent fiber tracts with opposite curvatures would develop maximum tension without motion. This process is connected with high energy consumption/demand and easily provokes myocardial ischemia [21].

There were patients with (*n* = 40) or without (n =16) prescribed treatment (newly diagnosed patients referred to our ambulatory clinic). Our group was mixed as concerned to therapy. After our study assessment, the therapy was introduced or improved especially in NYHA III/IV. The number of patients is too low to assess the relationship with RSC.

## 5. Limitations

The most important limitation of this study is the relatively small number of patients related to several exclusion criteria. Most of the patients have not met clinical indications for coronary angiography (young, without angina pectoris, and without risk factors for CAD). The risk for CAD was low; thus, coronary angiography was not indicated (unfortunately, multislice coronary computed tomography was not performed). The strategy of noninvasive identification of subgroups with a low likelihood of obstructive CAD is effective [22]. Slightly higher troponin might be not a result of myocardial ischemia; however, very detailed exclusion criteria and use of the high-sensitivity troponin I test may assure that troponin was released by ischemic event. There is a possible bias related to insufficient sample size. We were not able to calculate multivariable regression analysis.

The next problem related to the small number of patients is the fact that the value of NT-proBNP was elevated in both subgroups, but differences were on the border of statistical significance.

Thus, the result of this study is rather hypothesis-generating than causative. However, a study with a large group of patients might be able to achieve statistically significant differences.

## 6. Conclusion

Ischemia detected by an elevated troponin level in HCM patients might be strongly related to abnormal septal configuration. RSC as an unchangeable (during many years) feature seems to be an alerting signal of risk of ischemic events. The RSC subgroup was linked to a higher rate of nsVT, syncope, and family history of SCD.

## Figures and Tables

**Table 1:** Baseline characteristics of the patients.

NYHA	
Class I	6 (11%)
Class II	26 (46%)
Class III/IV	24 (43%)
CCS	
Class I	25 (45%)
Class II	20 (36%)
Class III/IV	11 (19%)
Syncope (*n*)	23 (41%)
Sudden death in family history (*n*)	22 (39%)
NSVT in Holter (*n*)	24 (43%)
EF (%)	62.5 ± 10.2
Mitral regurgitation trace	
Mild	40 (71%)
Moderate/severe	16 (29%)
Tricuspid regurgitation trace	
Mild	38 (68%)
Moderate/severe	18 (32%)
Systolic pulmonary artery pressure (mmHg)	35.4 ± 14.7
Maximum LV thickness (mm)	22.6 ± 4.9
Resting LVOT gradient ≥ 30 mmHg (*n*)	14
Provocable LVOT gradient ≥ 30 mmHg (*n*)	12
Left atrial diameter (cm), mean (SD)	4.89 ± 0.81

Data are presented as the number (percentage) or mean and standard deviation. CCS: Canadian Cardiovascular Society; EF: ejection fraction; LVOT: left ventricular outflow tract; LV: left ventricular; NSVT: nonsustained ventricular tachycardia; NYHA: New York Heart Association.

**Table 2:** Comparison of patients with reversed septal curvature and nonreversed septal curvature.

	RSC (*n* = 17)	Non-RSC (*n* = 39)	*p* value
hs-TnI (ng/l)	102 (29.2-214.7)	8.7 (5.3-18)	0.001
NT-proBNP (pg/ml)	1279 (367.3-1186)	551.7 (273-969)	0.056
NT-proBNP/ULN	6.51 ± 4.87	4.59 ± 4.23	0.14
LVOTG at rest (mmHg)	10 (6.47-52)	8.2 (5.4-21)	0.3
LVOTG provocable (mmHg)	51 (9.5-105)	13.6 (7.5-31)	0.04
STD (mm)	24.1 ± 5.3	19.8 ± 3.4	0.02
LVEDD (mm)	35.3 ± 6.6	42.1 ± 5.2	0.001
STD/LVEDD > 0.5	14/17 (82%)	9/39 (18%)	0.001
EF (%)	61.2 ± 9.8	63.4 ± 9.5	0.7
Systolic pulmonary artery pressure (mmHg)	38.3 ± 16.6	33.7 ± 13.5	0.3
nsVT	11/17 (65.7%)	13/39 (33.3%)	0.02
Syncope	11/17 (65%)	12/39 (31%)	0.02
Family history of SCD	10/17 (59%)	12/39 (31%)	0.04
Risk of SCD at 5 years (%)	8.83 (4.37-12.35)	3.09 (1.9-4.76)	0.002

Data are presented as the mean and standard deviation or median and interquartile range. EF: ejection fraction; LVEDD: left ventricular end-diastolic diameter; LVOTG: left ventricular outflow tract gradient; nsVT: nonsustained ventricular tachycardia; SCD: sudden cardiac death; STD: septal thickness at end diastole.

## Data Availability

All data used to support the findings of this study are available from the corresponding author upon request: Paweł Petkow Dimitrow, 2nd Department of Cardiology, Jagiellonian University Medical College, Jakubowskiego 2 Str., 30-688 Krakow, Poland, e-mail: dimitrow@mp.pl.
